# Case report: follow-up of long term prophylaxis of hereditary angioedema with an alternative attenuated androgenic drug

**DOI:** 10.1186/1939-4551-8-S1-A274

**Published:** 2015-04-08

**Authors:** João Tebyrica

**Affiliations:** 1 Unirio, Brazil

## Background

Hereditary Angioedema (HAE) is a disease caused by defective production or function of C1 inhibitor (C1-INH) and transmitted by autossomic dominant inheritance pattern. Treatment of HAE is divided into three parts: short-term and long-term prophylaxis, and treatment of acute attacks. The long-term prophylaxis of HAE is aimed at reducing the frequency and severity of acute attacks. This is usually made using attenuated androgens

## Methods

We report the case of four patients with HAE followed for more than 10 years, treated with oxymetholone, an attenuated androgen.

## Results

Four patients (one male; three female) with HAE have received oxymetholone 25 mg twice a week for long-term prophylactic treatment of HAE and have been followed for at least ten years. Two patients are well controlled and showed no acute attack in the last 3 years. One patient has allergic rhinitis and have had presented acute attacks associated with superior airways infections. One patient remained controlled for more than 20 years with oxymetholone but has shown repeated crises in the last two years with no apparent cause. Observed side effects were dyslipidemia and moderate weight gain in all patients. No hepatotoxicity were observed

## Discussion

Long –term prophylaxis of HAE is usually done with attenuated androgens. Danazol and stanozolol are the anabolic steroids most commonly used in clinical practice. Although the efficacy of these drugs in the treatment of HAE is well established, the mechanism for this effect remains unclear. There appears to be an enhancement of C1 INH protein production. Methyl-testosterone and oxymetholone were already described with same properties. In Brazil, stanozolol isn’t available and Oxymetholone is less expensive than danazol . All our four patients were from low income social status and oxymetolone were the only drug affordable for long-term use.

## Conclusion

Low doses of oxymetholone can be a reliable and safe attenuated androgen for patients with HAE when danazol or atanozolol are not available for long-term prophylaxis of HAE.

